# Cardiorespiratory fitness and physical activity and risk of SARS-CoV-2 and COVID-19 hospitalization: the HUNT study

**DOI:** 10.1186/s12879-026-12684-1

**Published:** 2026-01-27

**Authors:** Espen Alexander Eriksen, Javaid Nauman, Ulrik Wisløff, Torbjørn Omland, Dorthe Stensvold

**Affiliations:** 1 Division of Medicine and Laboratory Sciences, Institute of Clinical Medicine, Faculty of Medicine, University of Oslo, Oslo, Norway; 2 Department of Circulation and Medical Imaging, Faculty of Medicine and Health Sciences, Norwegian University of Science and Technology, Trondheim, Norway; 3 Zayed Center Health Sciences, Institute of Public Health, United Arab Emirates University, Al Ain, United Arab Emirates; 4 Division of Medicine, Department of Cardiology, Akershus University Hospital, Lørenskog, Norway

**Keywords:** COVID-19, Cardiorespiratory fitness, Physical activity, SARS-CoV-2, Hospitalization

## Abstract

**Background:**

Physical activity (PA) has been associated with a reduced risk of severe COVID-19 outcomes. However, the relationship between cardiorespiratory fitness (CRF) and the risk of SARS-CoV-2 infection and COVID-19 hospitalization has not been thoroughly investigated. We aimed to investigate the association of estimated CRF (eCRF) and leisure-time PA (LTPA) with risk of SARS-CoV-2 infection and COVID-19 related hospitalization in a general population of Norwegian adults.

**Methods:**

This cohort study included 48,821 adults participating in the population based Trøndelag Health Study (the HUNT Study). Individual data on pre-pandemic (2017–2019) eCRF and LTPA were linked to COVID-19 registries from February 2020 through September 2022. eCRF was categorized into sex-and-age specific quintiles based on V̇O_2peak_ (mL/kg/min) and LTPA was categorized based on metabolic equivalent hours per week (MET h/wk): inactive (0-3.5 MET h/wk), insufficiently active (> 3.5 to 7.5 MET h/wk), and sufficiently active (> 7.5 MET h/wk). Poisson regression was used to estimate incidence rate ratios (IRRs) and 95% confidence intervals (CIs) for the association of eCRF and LTPA with SARS-CoV-2 infection and COVID-19 hospitalization.

**Results:**

Age averaged 53.6 years (SD 16.8) and 53.9% were women. During 2.6 years of follow-up there were 5991 SARS-CoV-2 infections and 218 COVID-19 related hospitalizations. Fitness and LTPA categories did not associate with risk of infection. However, adults with the highest eCRF had significantly lower risk of hospitalization compared to adults with the lowest eCRF (IRR, 0.54, 95% CI, 0.34–0.86). Similarly, sufficiently active adults (> 7.5 MET h/wk) prior to the pandemic had significantly lower risk of being hospitalized compared to inactive adults (IRR, 0.60, 95% CI, 0.47–0.83).

**Conclusions:**

Higher eCRF and LTPA were not associated with risk of SARS-CoV-2 infection. In contrast, adults with high eCRF and LTPA were associated with a lower risk of COVID-19 related hospitalization compared to adults with low fitness and inactive lifestyles.

**Supplementary Information:**

The online version contains supplementary material available at 10.1186/s12879-026-12684-1.

## Background

Since its initial outbreak in March 2020, the severe acute respiratory syndrome coronavirus-2 (SARS-CoV-2) and the coronavirus disease 2019 (COVID-19) pandemic has caused more than 776 million confirmed cases of infections and more than 7 million deaths worldwide (as of 25th of September 2024) [1, 2]. In the U.S, the mean cost per inpatient stay of hospitalization increased by 26% (from 10,394 to 13,072 USD) from March 2020 to March 2022 [3]. Additionally, patients with more comorbidities required longer stays in the internal care units, increasing loss of productivity and thus inflicting indirect negative economic consequences both during and after the pandemic [4–7]. Norway managed better than the 3 other Nordic countries in terms of COVID-19 associated hospitalizations and deaths during the pandemic [8]. Nevertheless, by April 2022 as much as 25% of the entire Norwegian population had been infected (confirmed laboratory tests), 11,500 patients had been hospitalized and 2,600 COVID-19 related deaths had been reported [9, 10].

Cardiorespiratory fitness (CRF) and physical activity (PA) are well established and effective measures to reduce the risk of numerous chronic and non-communicable diseases, including premature death [11, 12]. Recent findings from cohort studies have indicated a potential dose-response relationship, where higher levels of PA and CRF appear to be associated with a reduced risk of severe COVID-19 outcomes and mortality within mixed adult populations [13–20]. However, studies examining this relationship has been performed in the early stages of the pandemic and include short follow-up time [19, 21]. In addition, there is a need to further elucidate the relationship between CRF levels with COVID-19 outcomes in larger and more general populations of adults.

Previous studies examining PA and CRF and COVID-19 outcomes have typically relied on single analytical approaches with limited handling of time-varying confounding, particularly for vaccination status [13, 15–17, 22–25]. Furthermore, the absence of methodological triangulation using multiple approaches with different underlying assumptions to address the same question has constrained causal interpretation. Therefore, the objective of this study was to investigate the association of pre-pandemic estimated CRF (eCRF) and leisure-time PA (LTPA) with risk of SARS-CoV-2 infection and COVID-19 related hospitalization, using multiple analytical approaches with different underlying assumptions, in a large and general population of adults. We hypothesized that higher pre-pandemic eCRF and LTPA levels were associated with lower risk of COVID-19 hospitalization.

## Methods

### Study design

This study was a prospective register-based cohort study and was conducted using the adult population in Nord-Trøndelag County, Norway (HUNT Study cohort). We utilized data from the most recent data collection (HUNT4), conducted between 29th of August 2017 and 23rd of February 2019 (pre-pandemic). All residents aged 20 and above were invited to participate in the study. Out of 103 800 invites, a total of 56,039 (54% of the invited population) participated [26]. Figure S1 (Supplementary Material) provides a list of complete cases included in our analyses after exclusion of participants due to missing values. Research ethics approval was given by the regional committee for Central Norway (REK# 479316). Participants provided written consent for their data to be shared with external researchers with REK-approvals to access the HUNT data material. We followed the Strengthening the Reporting of Observational Studies in Epidemiology (STROBE) reporting guidelines [27].

### COVID-19 registries

The Norwegian Surveillance System for Communicable Diseases (MSIS) [28], The Norwegian Intensive Care and Pandemic Registry (NIPaR) [29], The Norwegian Cause of Death Registry [30], and the Norwegian Immunization Registry [31] provided information regarding SARS-CoV-2 infections, COVID-19 related hospitalizations, deaths, and vaccination status, respectively, from 26th of February 2020 to 1st of October 2022 (2.6 years follow-up). The ICD-U07.1 code was used to confirm a positive PCR-test for SARS-CoV-2 in MSIS. NIPaR was the primary data source used in Norway for surveillance of new patients admitted to the hospital with COVID-19 incidence (ICD-U07.1), with a reported 92% data coverage [32]. The main cause of hospital admission was confirmed by the physician based on positive PCR-test for SARS-CoV-2 from MSIS [32, 33]. Registry data were merged with data from the HUNT Study using a unique personal identification number. Access to registry data was approved by the Norwegian Institute of Public Health.

### Cardiorespiratory fitness and physical activity

Assessments of eCRF and LTPA levels were performed using a previously validated questionnaire [34]. The questionnaire included three questions for quantifying the frequency, intensity and duration of LTPA. The frequency question, stated as “How often do you exercise?”, had five response categories: “never”, “less than once a week”, “once a week”, “two to three times a week”, and “almost every day”. The intensity question, stated as “How hard do you usually push yourself?”, had three response categories: “no sweating or heavy breathing”, “sweating and heavy breathing”, and “push myself to exhaustion”. The question on duration per session contained four response options: “less than 15 min”, “between 15 and 30 min”, “between 30 and 60 min”, and “more than 60 min”. Weighted values for each response were multiplied to calculate a PA index that has shown good correlation with objectively measured CRF [35]. To estimate CRF, we used a non-exercise prediction model from a previously derived and cross-validated subsample of healthy participants in HUNT 3 [35]. The sex-specific models included age, waist circumference, PA index, and resting heart rate. Participants were grouped based on age (10-year categories) and sex-specific quintiles of CRF based on the recommendations by Myers et al., in order to minimize the variability that inherently exists within different age groups and sexes in CRF [36]. CRF quintiles are represented by estimated peak oxygen uptake (V̇O_2peak_) in mL/kg/min: Q1: 28.3, 23.7, Q2: 35.0, 28.3, Q3: 38.7, 30.9, Q4: 42.0, 33.4, and Q5: 47.3, 37.1, for men and women, respectively. For LTPA, we converted the answers from the reported frequency, duration and intensity into metabolic equivalent hours per week (MET h/wk) categorized in three groups: inactive (0-3.5 MET h/wk), insufficiently active (> 3.5 to < 7.5 MET h/wk), and sufficiently active (> 7.5 MET h/wk), where the sufficiently active group corresponded to the minimal recommended amount of PA from the World Health Organization´s PA guidelines of 150 min of moderate-to-high intensity PA per week [37].

### Statistical analysis

Our primary outcomes were risk of SARS-CoV-2 infection and hospitalization due to COVID-19. The number of deaths observed during follow-up within the studied cohort was insufficient to establish a risk estimate based on eCRF and LTPA as exposure and only allowed us to merge the reported deadly cases with reported infection cases (see Supplementary). We used modified Poisson regression with a log link and person-time offset to estimate incidence rate ratios (IRRs) and 95% confidence intervals (CIs). This approach is mathematically equivalent to Cox regression for estimating rate ratios while providing direct interpretation of incidence rates [38, 39]. We examined the association of SARS-CoV-2 infection and COVID-19 hospitalization by eCRF in quintiles (Q2-Q5 vs. Q1 (reference), “Fit” (> 20%) vs. “Least fit” (< 20%, reference), and per unit increase in 1 MET (3.5 mL/kg/min) and per unit increase of 1 mL/kg/min in eCRF. For LTPA levels, each of the 2 upper LTPA categories were compared to the lowest category (reference). We also performed analysis by merging the two lower categories into one category (insufficiently active, reference) and compared it to the sufficiently active group (Supplementary Material). Model 1 was adjusted for demographic characteristics (age, sex, education, income), including lifestyle factors such as alcohol and smoking, and model 2 added comorbidities such as hypertension, myocardial infarction, angina, heart failure, stroke and cancer. Furthermore, we performed subgroup analyses to check for the modification of the association of both pre-pandemic eCRF and LTPA by sex, BMI, smoking, income, hypertension, diabetes, myocardial infarction and cancer. Demographic variables, lifestyle factors and comorbidities were self-reported in HUNT4.

Because vaccination status changed during follow-up, we modeled vaccination as a time-dependent covariate using piecewise Poisson regression, splitting follow-up at vaccination dates. This approach is equivalent to Cox regression with time-varying covariates. We also conducted secondary analyses using Cox proportional hazards models with counting-process notation and flexible parametric survival models (stpm2) for additional validation. We also performed competing risks analyses using cause-specific hazards and Fine-Gray subdistribution models treating death as a competing event. Additional sensitivity analyses were performed treating vaccination as a competing event (with appropriate caution regarding interpretation), restricting to participants with exposure assessments in 2018–2019, multiple imputation for missing covariates (13% missing), and testing for nonlinearity using restricted cubic splines. To assess potential residual confounding, we conducted a post-hoc negative control outcome analysis using accidental mortality during follow-up [40]. We obtained information on underlying cause of death from the Norwegian Cause of Death Registry, and accidental deaths were defined according to ICD-10 codes V00-X59. All analyses were conducted using STATA. All tests were two-sided with a statistical significance level at alpha 0.05.

## Results

### Characteristics at baseline

Characteristics of the participants according to pre-pandemic eCRF are presented in Table 1. The mean age of participants were 53.6 years (SD 16.8), and 53.9% were women. Compared to participants with low eCRF (Q1), those with the highest eCRF (Q5) had lower prevalence of obesity (0.7% vs. 74.2%), current smokers (6.3% vs. 12.5%), and co-morbid conditions such as hypertension (26.2% vs. 48.0%) and diabetes (4.1% vs. 14.8%), and were more educated (≥ 12 years: 53.9% vs. 30.2%). Similarly, sufficiently active participants had lower prevalence of obesity (18.5% vs. 31.5%), current smokers (6.1% vs. 14.9%), and diabetes (6.6% vs. 9.7%), and were more educated (≥ 12 years: 48.2% vs. 32.1%), compared with inactive participants (Table S1). During 2.6 years of follow-up, there were 5991 reported incident cases of SARS-CoV-2, 218 hospitalizations and 4 deaths due to COVID-19.

**Table 1:** Characteristics of participants according to pre-pandemic estimated cardiorespiratory fitness

Characteristics	All (*n* = 48,821)	eCRF Q1 (*n* = 9,698)	eCRF Q2 (*n* = 9,728)	eCRF Q3 (*n* = 9,772)	eCRF Q4 (*n* = 9,766)	eCRF Q5 (*n* = 9,857)
Age, years, mean (SD)	53.6 (16.8)	54.4 (17.1)	53.9 (16.9)	53.6 (16.8)	53.3 (16.7)	52.7 (16.5)
Women, n (%)	26,297 (53.9)	5219 (53.8)	5227 (53.7)	5271 (53.9)	5258 (53.8)	5322 (53.9)
BMI, kg/m^2^, n (%)						
< 18.5	470 (1.0)	1 (0.01)	7 (0.07)	62 (0.63)	151 (1.55)	249 (2.5)
18.5–24.9	15,946 (32.7)	147 (1.5)	1162 (11.9)	2926 (29.9)	4711 (48.2)	7000 (71.0)
25.0-29.9	20,615 (42.2)	2360 (24.3)	5582 (57.4)	5595 (57.3)	4536 (46.5)	2542 (25.8)
≥ 30.0	11,790 (24.2)	7190 (74.2)	2977 (30.6)	1189 (12.2)	368 (3.8)	66 (0.7)
Years of education, n (%)						
< 10 years	12,324 (25.2)	3091 (31.9)	2694 (27.7)	2539 (26.0)	2250 (23.0)	1750 (15.8)
10–12 years	16,629 (34.1)	3681 (38.0)	3553 (36.5)	3417 (35.0)	3183 (32.6)	2795 (28.4)
≥ 12 years	19,868 (40.7)	2926 (30.2)	3481 (35.8)	3816 (39.0)	4333 (44.4)	5312 (53.9)
Annual income, NOK, n (%)						
< 450,000	14,602 (29.9)	3668 (37.8)	3114 (32.0)	2848 (29.1)	2726 (27.9)	2246 (22.8)
450,000–1 million	25,223 (51.7)	4590 (51.0)	5056 (52.0)	5146 (52.7)	5063 (51.8)	5008 (50.8)
> 1 million	8996 (18.4)	1080 (11.1)	1558 (16.0)	1778 (18.2)	1977 (20.2)	2603 (26.4)
Smoking status, n (%)						
Never	21,618 (44.3)	3677 (37.9)	3898 (40.1)	4278 (43.8)	4575 (46.9)	5190 (52.7)
Current	4834 (9.9)	1216 (12.5)	1105 (11.4)	1008 (10.3)	885 (9.1)	620 (6.3)
Former	22,369 (45.8)	4805 (49.5)	4725 (48.6)	4486 (45.9)	4306 (44.1)	4047 (41.1)
Alcohol units, n (%)						
0 to < 7	35,827 (73.4)	7522 (77.6)	7214 (74.2)	7085 (72.5)	7028 (72.0)	6978 (70.8)
7 to ≤ 14	10,122 (20.7)	1612 (16.6)	1939 (19.9)	2098 (21.5)	2162 (22.1)	2311 (23.5)
> 14	2872 (5.9)	564 (5.8)	575 (5.9)	589 (6.0)	576 (5.9)	568 (5.8)
Comorbidities, n (%)						
Hypertension	17,634 (36.1)	4655 (48.0)	3904 (40.1)	3404 (34.8)	3093 (31.7)	2578 (26.2)
Diabetes	3963 (8.1)	1434 (14.8)	886 (9.1)	703 (7.2)	540 (5.5)	400 (4.1)
Cancer	3566 (7.3)	729 (7.5)	757 (7.8)	692 (7.1)	722 (7.4)	666 (6.8)
Myocardial infarction	1653 (3.4)	382 (3.9)	375 (3.9)	353 (3.6)	293 (3.0)	250 (2.5)
Angina	1248 (2.6)	282 (2.9)	267 (2.7)	254 (2.6)	242 (2.5)	203 (2.1)
Stroke	1397 (2.9)	335 (3.5)	317 (3.3)	272 (2.8)	255 (2.6)	218 (2.2)
Heart failure	657 (1.4)	166 (1.7)	160 (1.6)	110 (1.1)	118 (1.2)	103 (1.0)

Data are presented as number (percentage) of participants unless stated otherwise. Abbreviations: BMI, body mass index; eCRF Q1-Q5, estimated cardiorespiratory fitness in quintiles based on sex and age. Alcohol Units, (12.8 g alcohol) consumed over a 2-week period

### Risk of SARS-CoV-2 infection

Participants with higher eCRF (Q2-Q5) did not have a lower risk of testing positive for SARS-CoV-2 compared to participants with low pre-pandemic eCRF (Q1), (Table 2). Further, there was no association between pre-pandemic LTPA and risk of testing positive for SARS-CoV-2 (Table 2).

**Table 2:** Risk of SARS-CoV-2 infection by pre-pandemic estimated cardiorespiratory fitness and leisure-time physical activity

	COVID-19 infections	IRR (95% CI)	
eCRF ^a^	Number	Model 1	Model 2
Q1	1155	1.00 (ref)	1.00 (ref)
Q2	1182	1.00 (0.93–1.08)	0.99 (0.92–1.06)
Q3	1197	1.00 (0.93–1.07)	0.98 (0.91–1.06)
Q4	1210	1.00 (0.93–1.07)	0.98 (0.91–1.05)
Q5	1247	0.99 (0.93–1.07)	0.97 (0.90–1.04)
Per mL/kg/min	5991	1.00 (0.99-1.00)	0.99 (0.99–1.00)
**Least fit (≤ 20%)**	1155	1.00 (ref)	1.00 (ref)
**Fit (> 20%)**	4836	0.99 (0.94–1.06)	0.98 (0.92–1.04)
**Leisure-time physical activity**			
Inactive ^b^	2347	1.00 (ref)	1.00 (ref)
Insufficiently active ^c^	756	0.98 (0.90–1.05)	0.98 (0.91–1.05)
Sufficiently active ^d^	2888	0.97 (0.93–1.02)	0.97 (0.92–1.02)
Per MET	5991	0.99 (0.98–1.01)	0.99 (0.98–1.00)

Abbreviations: IRR, incidence rate ratio; CI, confidence interval; MET, Metabolic equivalent of task. ^a^ Quintiles of VO_2peak_ in mL/kg/min for men and women, respectively: Q1: 28.2 and 23.7; Q2: 35.0 and 28.3; Q3: 38.7 and 30.9; Q4: 42.0 and 33.4; Q5: 47.3 and 37.1. ^b^ = 0 to 3.5 MET h/wk; ^c^ = more than 3.5 to less than 7.5 MET h/wk; ^d^ = 7.5 or more MET h/wk. Model 1: Adjusted for age, sex, education, income, smoking, alcohol. Model 2: Adjusted for age, sex, education, income, smoking, alcohol, diabetes, hypertension, myocardial infarction, angina, heart failure, stroke, and cancer

### Risk of hospitalization

As shown in Table 3, higher eCRF was associated with lower risk of being hospitalized, where the participants in the two highest quintiles of eCRF (Q4; 42.0 and 33.4 mL/kg/min for men and women, respectively, and Q5; 47.3 and 37.1 mL/kg/min, for men and women, respectively) had significantly lower risk of being hospitalized for COVID-19 compared to individuals in the lowest eCRF group, (IRR, 0.66, 95% CI, 0.44–0.99 for Q4, and, IRR, 0.54, 95% CI, 0.34–0.86 for Q5) (Table 3). When grouping participants into “Fit” (> 20%; >28.2 and > 23.7 mL/kg/min, for men and women, respectively) and “Least Fit” (< 20%; <28.2 and < 23.7 ml/kg/min, for men and women, respectively), the Fit group had a 34% lower risk of being hospitalized for COVID-19 (IRR: 0.66, 95% CI, 0.49–0.89) compared to the Least Fit group. Every 1 MET (3.5 mL/kg/min) increase in eCRF was associated with 15% lower risk of being hospitalized due to COVID-19 (IRR: 0.85, 95% CI, 0.79–0.92), while a fitness increase of 1 mL/kg/min was associated with a lower risk by 4% (IRR: 0.96. 95% CI, 0.93–0.98). Also, participants reporting to be sufficiently active prior to the pandemic had significantly lower risk of being hospitalized for COVID-19 compared with those who were inactive (IRR: 0.60, 95% CI, 0.47–0.83) (Table 3). The insufficiently active group had no significant risk reductions of being hospitalized for COVID-19 compared to the inactive group (IRR, 0.83, 95% CI, 0.57–1.20). Including the 4 reported deaths in our analysis did not alter the estimates substantially (Supplementary Table S2 & S3).

**Table 3:** Risk of hospitalization due to COVID-19 by pre-pandemic estimated cardiorespiratory fitness and leisure-time physical activity

	COVID-19 hospitalizations	IRR (95% CI)	
eCRF ^a^	Number	Model 1	Model 2
Q1	66	1.00 (ref)	1.00 (ref)
Q2	45	0.71 (0.49–1.04)	0.72 (0.49–1.05)
Q3	40	0.66 (0.45–0.99)	0.68 (0.46–1.02)
Q4	38	0.64 (0.43–0.96)	0.66 (0.44–0.99)
Q5	29	0.51 (0.33–0.81)	0.54 (0.34–0.85)
Per mL/kg/min	218	0.95 (0.93–0.97)	0.96 (0.93–0.98)
**Least fit (≤ 20%)**	66	1.00 (ref)	1.00 (ref)
**Fit (> 20%)**	152	0.64 (0.48–0.86)	0.66 (0.49–0.89)
**Leisure-time physical activity**			
Inactive ^b^	113	1.00 (ref)	1.00 (ref)
Insufficiently active ^c^	37	0.82 (0.56–1.20)	0.83 (0.57–1.20)
Sufficiently active ^d^	68	0.58 (0.42–0.79)	0.60 (0.44–0.82)
Per MET	218	0.84 (0.78–0.91)	0.85 (0.79–0.92)

Abbreviations: IRR, incidence rate ratio; CI, confidence interval; MET, Metabolic equivalent of task. ^a^ Quintiles of VO_2peak_ in mL/kg/min for men and women, respectively: Q1: 28.2 and 23.7; Q2: 35.0 and 28.3; Q3: 38.7 and 30.9; Q4: 42.0 and 33.4; Q5: 47.3 and 37.1. ^b^ = 0 to 3.5 MET h/wk; ^c^ = more than 3.5 to less than 7.5 MET h/wk; ^d^ = 7.5 or more MET h/wk. Model 1: Adjusted for age, sex, education, income, smoking, alcohol. Model 2: Adjusted for age, sex, education, income, smoking, alcohol, diabetes, hypertension, myocardial infarction, angina, heart failure, stroke, and cancer

### Subgroup- and sensitivity analysis

**Fig. 1:**
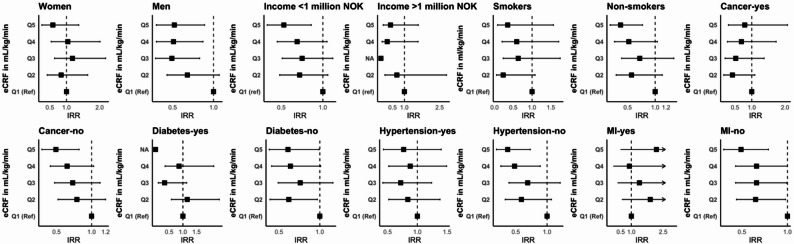
Association between cardiorespiratory fitness and COVID-19 hospitalization in selected subgroups. Abbreviations: IRR, Incidence rate ratios (black squares), 95% CIs (horizontal lines), right pointing arrows indicate CIs exceeding 3.0, MI; myocardial infarction. IRRs are adjusted for age, sex, body mass index, education, income, smoking, alcohol, diabetes, hypertension, MI, heart failure, stroke and cancer

Subgroup analyses revealed consistent protective effects across sex, income, smoking status, and most comorbidities, except a significant effect modification by diabetes status (P-interaction = 0.02) (Fig. 1, Table S4). While non-diabetic participants demonstrated protective effects across all eCRF quintiles, diabetic participants showed no significant associations. Furthermore, protective associations between LTPA and COVID-19 hospitalization were consistent across all subgroups of participants (Table S5).

We also conducted a sensitivity analysis by excluding participants with co-morbid conditions (history of myocardial infarction, angina, stroke, heart failure or cancer) (Table S6 and S7). Here we found an even lower risk of being hospitalized among participants with the highest eCRF (Q5; 47.3 and 37.1 mL/kg/min, for men and women, respectively) compared to participants with the lowest eCRF (Q1: 28.2 and 23.7, for men and women, respectively) (IRR: 0.44, 95% CI, 0.24–0.80) (Table S6). Similarly, for LTPA, we found an even lower risk of being hospitalized among those sufficiently active compared to the inactive group (IRR: 0.55, 95% CI, 0.37–0.82) (Table S7).

Results from additional analyses demonstrated the robustness of our primary findings. Time-to-event approaches including piecewise Poisson, time-dependent Cox, and flexible parametric survival models handling vaccination as a time-dependent covariate yielded estimates consistent with our primary Poisson regression results (Supplementary Tables S8–S9, S10–S11).

Competing risks analyses treating death as a competing event produced similar inferences using both cause-specific hazards and Fine-Gray subdistribution models (Table S12). When treating vaccination as a competing event, we observed expected descriptive differences in effect sizes, but the fundamental association between higher pre-pandemic eCRF and lower COVID-19 hospitalization risk remained unchanged (Table S13). Additional sensitivity analyses confirmed the stability of our findings: restricting to participants with recent exposure assessments (2018–2019) did not materially alter the associations (Table S14), and standardized absolute risk measures complemented the relative risk estimates (Table S15). Additionally, sensitivity analyses using restricted cubic splines (*p* > 0.25) and fractional polynomials (*p* > 0.79) confirmed an approximately linear relationship, and more granular categorization approaches yielded results consistent with our primary analysis (data not shown).

During follow-up, 39 deaths were classified as accidental according to the Norwegian Cause of Death Registry. Pre‑pandemic eCRF and LTPA showed no clear associations with the risk of accidental death. Compared to the lowest quintile of eCRF, the IRR was 0.81 (95% CI, 0.30–2.21) for highest eCRF quintile. Similarly, sufficiently active individuals showed no protective effect for accidental mortality compared to inactive individuals (IRR 0.76, 95% CI, 0.36–1.60) (Table S16). Adjusted Cox proportional models produced hazard ratios similar to the Poisson IRRs (Table S17).

## Discussion

In this prospective, register-based cohort study, we observed that pre-pandemic eCRF and LTPA levels were not associated with risk of being infected by SARS-CoV-2. However, participants with high eCRF and sufficient LTPA levels had 46% and 40% lower risk of COVID-19 hospitalization, respectively, compared to those with low eCRF and low LTPA levels. The results remained consistent after adjusting for multiple covariates.

### Risk of infection

Several studies have reported an association between higher levels of PA and lower risk for infectious diseases [19, 20, 25, 39, 41]. It has been suggested that PA and exercise reduces concentrations of inflammatory cytokines and reduces activity of the inflammasome, and thereby improve immune health [42]. In line with this, a recently published paper by Muñoz-Vergara et al. [15] observed a reduced risk of SARS-CoV-2 infection among older adults (mean 75.7 years) with > 7.5 MET/h/wk compared to their inactive counterparts. In contrast, no association between PA levels and risk of SARS-CoV-2 infection were found in the present study. This inconsistency is likely attributed to several methodological differences. In Muñoz-Vergara et al., infection rate was determined using questionnaires, making it very likely that individuals who did not exhibit any symptoms, despite being infected, were neither tested nor counted for. Thus, the association seen in Muñoz-Vergara et al. is probably influenced by the underestimation of SARS-CoV-2 infection, especially among asymptomatic individuals. In the present study, data on SARS-CoV-2 infection was provided by the Norwegian immunization registry. As Norway conducted extensive testing of both symptomatic and asymptomatic individuals, our study represents a more nuanced and accurate estimate of the association between PA and SARS-CoV-2 infection.

Few studies have investigated the relationship between CRF and SARS-CoV-2 infection. In 2021, Christensen et al. [13] reported no reduced risk among older adults with high eCRF from the UK Biobank cohort (*n* = 2690, median age 70 years), during the first 4 months of the pandemic [13]. Similarly, our results support the findings of Christensen et al. showing no relationship between eCRF and SARS-CoV-2 infection rate in a much larger population.

### Risk of hospitalization

Participants who reported to be sufficiently active had a 40% lower risk of being hospitalized compared to those who were inactive in the present study. Thus, our data support previous findings showing that moderate to high PA levels are associated with significantly lower risk of severe COVID-19 outcomes compared to physically inactive individuals [15, 20, 25, 39]. Further, we observed an inverse relationship between eCRF and risk of hospitalization, where participants with the highest eCRF had a 46% lower risk of being hospitalized compared to the least fit participants. Importantly, we observed a significant 4% risk reduction for every 1 mL/min/kg increase in eCRF. Thus, our data confirms previous findings within more selected populations, reporting a significant 32% and 23% lower risk of COVID-19-related hospitalization in high fit versus low fit US veterans and patients at a private health care clinic (*n* = 246), respectively [16, 17]. Furthermore, our analysis was strengthened by the calculation of standardized absolute risk estimates from cumulative incidence functions, including risk differences and risk ratios, which complement the IRRs and enhance the clinical interpretability of the substantial risk reduction for hospitalization observed with higher levels of eCRF and LTPA. Additionally, it has previously been suggested an augmented effect of COVID-19 vaccines on the immune system with increased PA and CRF levels [42, 43]. However, our sensitivity analyses adding COVID-19 vaccination status as a time-varying covariate did not affect the risk estimates for eCRF and PA and its association with risk of COVID-19 hospitalization.

### Strengths and limitations

This study has some strengths. The large cohort, with a balanced sex distribution, makes our findings more transferable to a wider range of the general population of adults. The adjustments of several well-established risk factors for COVID-19 [44] and comorbidities in our analyses enabled robust and precise estimates. However, several limitations in this study warrant considerations. Assessment of LTPA were self-reported and may have led to misclassification, which further could have underestimated the true association. Furthermore, the questionnaire only assessed LTPA, thus, household activities and occupational PA were missing, which may have influenced the magnitude of the true association [45]. Importantly, the public health authorities in Norway introduced unique approaches to mitigate the spread of the virus, including one of the strictest lockdowns and one of the highest percentages of a population being tested [46]. The Norwegian Institute of Public Health began testing for COVID-19 as early as January 23rd, 2020, detecting the first case of SARS-CoV-2 on 26th of February [47]. However, significant testing constraints due to global shortage in testing reagents and equipment occurred from March 2020 through August 2020 [48]. This shortage was later alleviated by a new PCR test developed by NTNU which were scaled up for nationwide use from September 2020 [49]. Thus, we acknowledge that our results may be subject to surveillance bias due to under ascertainment of positive cases in the initial phase of the pandemic.

Norway´s vaccination program was launched in late December 2020, and by June 2022 an estimated 80.5% of the population had received their first vaccine dose, 75.1% had completed their second dose, and 55.9% had received at least one booster dose [50]. The vaccine uptake was particularly high among adults, reaching approximately 90% by July 2021 [51]. These comprehensive measures likely altered infection rates and led to relatively low rates of severe events related to COVID-19 [8]. In our main analyses, vaccination status was treated as a time-varying covariate to account for the protection conferred by vaccination. Additionally, we performed competing-risk sensitivity analyses treating vaccination as a competing event. Nevertheless, these subdistribution hazard ratios should be interpreted as descriptive effects on cumulative incidence in the presence of vaccine rollout rather than etiologic effects, given potential selection issues related to fitness levels influencing vaccination timing. Finally, although we implemented a negative control outcome analysis using accidental mortality, this analysis was limited by the relatively small number of events, resulting in imprecise estimates centered around the null. The lack of a strong association between pre‑pandemic eCRF/LTPA and accidental death provides some reassurance that our main results are not entirely driven by a general pattern of unmeasured confounding affecting all outcomes. However, this analysis cannot exclude more modest residual confounding. Moreover, we could not address exposure measurement error in eCRF and LTPA, nor selection and survival processes, with the available data. Thus, while our findings are robust across several conventional time‑to‑event and competing risk models, they should not be interpreted as definitive causal effects. Future work using triangulated causal approaches, including negative control exposures and outcomes, quantitative bias analyses, within‑family comparisons, and quasi‑experimental designs, will be important to further clarify the causal impact of fitness and LTPA on severe COVID‑19 outcomes and pandemic preparedness.

## Conclusion

In this general population of adults, high eCRF and sufficient LTPA levels prior to the pandemic were not associated with a reduced risk of SARS-CoV-2 infection. However, participants with sufficient LTPA and moderate to high levels eCRF had a substantially lower risk of COVID-19 hospitalization. Because of the observational design and the possibility of residual confounding and measurement error, the observed associations should not be interpreted as definitive causal effects. However, our findings are consistent with the interpretation that better fitness and higher PA may reduce severe COVID-19 risk and support ongoing efforts to promote PA and fitness as part of broader strategies to improve population health and resilience against respiratory infections. Future research using dedicated causal methods could strengthen evidence for targeted public‑health interventions.

## Supplementary Information

Below is the link to the electronic supplementary material.

Supplementary Material

## Data Availability

The datasets used for analysis in this study may be made available by separate applications to HUNT Research center at https://www.ntnu.edu/hunt, and the Norwegian Institute of Public Health at https://helsedata.no/en/.
