# Supplementary material for: Cardiorespiratory fitness and physical activity and risk of SARS-CoV-2 and COVID-19 hospitalization: the HUNT study
Source: BMC Infect Dis. 2026 Jan 27;26:396. doi: 10.1186/s12879-026-12684-1 (PMC12918651; doi:10.1186/s12879-026-12684-1)
Supplement: Supplementary file 1 — Supplementary Material [file 12879_2026_12684_MOESM3_ESM.docx]

**Supplementary material**

**Figure S1.** Flow chart - Exclusion of participants due to missing values

**Table S1**. Characteristics of participants according to pre-pandemic baseline leisure-time physical activity levels

**Table S2**. Adjusted Incidence Rate Ratio of Hospitalization and Death due to COVID-19 by Pre-Pandemic Estimated Fitness Levels

**Table S3**. Adjusted Incidence Rate Ratio of Hospitalization and Deaths due to COVID-19 by Level of Pre-Pandemic leisure-time Physical Activity

**Table S4.** Adjusted Incidence Rare Ratio of Hospitalization Due to COVID-19 by Pre-Pandemic Estimated Fitness: Subgroup Analysis.

**Table S5.** Adjusted Incidence Rate Ratio of Hospitalization Due to COVID-19 by Level of Pre-Pandemic Physical Activity Level, Subgroup Analysis.

**Table S6**. Adjusted Incidence Rate Ratio of Hospitalization due to COVID-19 by Pre-Pandemic estimated fitness levels, after excluding individuals with co-morbid conditions

**Table S7**. Adjusted Incidence Rate Ratio of Hospitalization due to COVID-19 by level of Pre-Pandemic Physical Activity, after excluding individuals with co-morbid conditions

**Table S8.** Adjusted Incidence Rate Ratio of Hospitalization due to COVID-19 by Pre-Pandemic estimated fitness levels, additionally adjusting for SARS CoV-2 vaccination status (modelled as time-dependent)

**Table S9.** Adjusted Incidence Rate Ratio of Hospitalization due to COVID-19 by level of Pre-Pandemic Physical Activity, additionally adjusting for SARS CoV-2 vaccination status (modelled as time-dependent)

**Table S10.** Hazard Ratios from Cox model for Hospitalization due to COVID-19 by Pre-Pandemic Estimated Cardiorespiratory Fitness and Physical Activity.

**Table S11.** Hazard Ratios from Flexible Parametric Model (stpm2) for Hospitalization due to COVID-19 by Pre-Pandemic Estimated Cardiorespiratory Fitness and Physical Activity.

**Table S12**. Competing Event (Death) Analyses for Hospitalization due to COVID-19 by Pre-Pandemic Estimated Cardiorespiratory Fitness and Physical Activity.

**Table** **S13**. Competing Event (vaccination) Analyses for Hospitalization due to COVID-19 by Pre-Pandemic Estimated Cardiorespiratory Fitness and Physical Activity.

**Table S14**. Restricting to recent exposure keeping 01 Jan 2018 onward

**Table S15**. Standardized Cumulative Incidence (CIF) of COVID-19 hospitalization by Pre-Pandemic Estimated Cardiorespiratory fitness, at end of follow-up.

**Table S16**. Association between accidental death and pre-pandemic estimated cardiorespiratory fitness and leisure-time physical activity.

**Table S17**. Hazard Ratios from Cox Model for Accidental Deaths by Pre-Pandemic Estimated Cardiorespiratory Fitness and Physical Activity.

The supplementary material provides additional information about the authors’ results and work for the readers.

HUNT4 (2017-2019)

n=56,039

7218 excluded

5154 Missing physical activity data

17 Missing data on body mass index

170 Missing data on education

1089 Missing data on income

132 Missing data on smoking

656 Missing data on alcohol

48,821 participants

(26,297 female, 22,524 male)

**Figure S1.** Exclusion of participants due to missing values.

**Table S1.** Characteristics of participants according to pre-pandemic baseline physical activity levels

| Characteristics | All (n=48,821) | Inactive (n=18,282) | Insufficiently active (n=6954) | Sufficiently active (n=23,585) |
| --- | --- | --- | --- | --- |
| Age, years, mean (SD) | 53.6 (16.8) | 53.2 (17.3) | 56.8 (16.8) | 52.8 (16.3) |
| Women, n (%) | 26,297 (53.9) | 9083 (49.7) | 4013 (57.7) | 13,201 (56.0) |
| Body mass Index, kg/m^2^, n (%) |  |  |  |  |
| <18.5 | 470 (1.0) | 217 (1.2) | 69 (1.0) | 184 (0.8) |
| 18.5-24.9 | 15,946 (32.7) | 4765 (26.1) | 2232 (32.1) | 8949 (37.9) |
| 25.0-29.9 | 20,615 (42.2) | 7544 (41.2) | 2971 (42.7) | 10,100 (42.8) |
| ≥30.0 | 11,790 (24.1) | 5756 (31.5) | 1682 (24.2) | 4352 (18.5) |
| Years of education, n (%) |  |  |  |  |
| <10 years | 12,324 (25.2) | 5646 (30.9) | 2024 (29.1) | 4654 (19.7) |
| 10-12 years | 16,629 (34.1) | 6770 (37.0) | 2289 (32.9) | 7570 (32.1) |
| ≥12 years | 19,868 (40.7) | 5866 (32.1) | 2641 (38.0) | 11,361 (48.2) |
| Annual income, NOK, n (%) |  |  |  |  |
| <450,000 | 14,602 (29.9) | 6356 (34.8) | 2413 (34.7) | 5833 (24.7) |
| 450,000-1 million | 25,223 (51.7) | 9278 (50.7) | 3481 (50.1) | 12,464 (52.9) |
| >1 million | 8996 (18.4) | 2648 (14.5) | 1060 (15.2) | 5288 (22.4) |
| Smoking status, n (%) |  |  |  |  |
| Never | 21,618 (44.3) | 7384 (40.4) | 3014 (43.3) | 11,220 (47.6) |
| Current | 4834 (9.9) | 2735 (14.9) | 665 (9.6) | 1434 (6.1) |
| Former | 22,369 (45.8) | 8163 (44.7) | 3275 (47.1) | 10,931 (46.3) |
| Alcohol status, n (%) |  |  |  |  |
| 0 to <7 | 35,827 (73.4) | 13,811 (75.5) | 5260 (75.6) | 16,756 (71.0) |
| 7 to ≤14 | 10,122 (20.7) | 3384 (18.5) | 1342 (19.3) | 5396 (22.9) |
| >14 | 2872 (5.9) | 1087 (6.0) | 352 (5.1) | 1433 (6.1) |
| Comorbidities, n (%) |  |  |  |  |
| Hypertension | 17,634 (36.1) | 6956 (38.1) | 2872 (41.3) | 7806 (33.1) |
| Diabetes | 3963 (8.1) | 1769 (9.7) | 638 (9.2) | 1556 (6.6) |
| Cancer | 3566 (7.3) | 1296 (7.1) | 603 (8.7) | 1667 (7.1) |
| Myocardial infarction | 1653 (3.4) | 735 (4.0) | 250 (3.4) | 668 (2.8) |
| Angina | 1248 (2.6) | 567 (3.1) | 206 (3.0) | 475 (2.0) |
| Stroke | 1397 (2.9) | 544 (3.0) | 232 (3.3) | 621 (2.6) |
| Heart failure | 657 (1.4) | 296 (1.6) | 117 (1.7) | 244 (1.0) |

Data are presented as number (percentage) of participants unless stated otherwise. Abbreviations: BMI, body mass index; eCRF Q1-Q5, estimated cardiorespiratory fitness in quintiles based on sex and age, Alcohol Units; (12.8 g alcohol) consumed over a 2-week period.

**Table S2.** Adjusted Incidence Rate Ratio of Hospitalization and Death Due to COVID-19 by Pre-Pandemic Estimated Fitness Levels

| **eCRF** | **Hospitalization + Deaths** | **IRR (95% CI)** | |
| --- | --- | --- | --- |
|  |  | Model 2 | Model 3 |
| Q1 | 67 | 1.00 (ref) | 1.00 (ref) |
| Q2 | 47 | 0.73 (0.50 – 1.06) | 0.73 (0.50 – 1.07) |
| Q3 | 40 | 0.65 (0.44 – 0.97) | 0.67 (0.45 – 0.99) |
| Q4 | 39 | 0.64 (0.43 – 0.96) | 0.66 (0.44 – 0.99) |
| Q5 | 29 | 0.50 (0.32 – 0.79) | 0.53 (0.33 – 0.83) |
|  |  |  |  |
| Least fit (≤20%) | 67 | 1.00 (ref) | 1.00 (ref) |
| Fit (>20%) | 155 | 0.64 (0.48 – 0.86) | 0.66 (0.49 – 0.89) |
|  |  |  |  |
| Per MET | 222 | 0.84 (0.78 – 0.91) | 0.85 (0.78 – 0.92) |
| Per mL/kg/min | 222 | 0.95 (0.93 – 0.97) | 0.95 (0.93 – 0.98) |

Abbreviations: IRR, incidence rate ratio; CI, confidence interval. Q1: 28.2 for men and 23.7 for women Q2: 35.0 for men and 28.3 for women Q3: 38.7 for men and 30.9 for women Q4: 42.0 for men and 33.4 for women, Q5: 47.3 for men and 37.1 for women. Model 1: Adjusted for age, sex, education, income, smoking, alcohol. Model 2: Adjusted for age, sex, education, income, smoking, alcohol, diabetes, hypertension, myocardial infarction, angina, heart failure, stroke, and cancer.

**Table S3.** Adjusted Incidence Rate Ratio of Hospitalization and Deaths Due to COVID-19 by Level of Pre-Pandemic Physical Activity

| **Physical activity** | **Hospitalization + Deaths** | **IRR (95% CI)** | |
| --- | --- | --- | --- |
| **3 categories^a^** |  | Model 1 | Model 2 |
| Inactive | 114 | 1.00 (ref) | 1.00 (ref) |
| Insufficiently active | 39 | 0.86 (0.59 – 1.25) | 0.86 (0.60 – 1.25) |
| Sufficiently active | 69 | 0.59 (0.43 – 0.80) | 0.60 (0.44 – 0.82) |
| **2 categories^b^** |  |  |  |
| Insufficiently active | 153 | 1.00 (ref) | 1.00 (ref) |
| Sufficiently active | 69 | 0.61 (0.46 – 0.82) | 0.63 (0.47 – 0.84) |

Abbreviations: MET, Metabolic equivalent of task; IRR, incidence rate ratio; ^a^Inactive = 0 to 3.5 MET h/wk; insufficiently active = more than 3.5 to less than 7.5 MET hr/wk; and sufficiently active = 7.5 or more MET h/wk; ^b^Insufficiently active = 0 to less than 7.5 MET hr/wk; and sufficiently active 7.5 or more MET h/wk. Model 1: Adjusted for age, sex, body mass index, education, income, smoking, alcohol. Model 2: Adjusted for age, sex, body mass index, education, income, smoking, alcohol, diabetes, hypertension, myocardial infarction, angina, heart failure, stroke, and cancer

**Table S4**. Adjusted Incidence Rate Ratio of Hospitalization Due to COVID-19 by Pre-Pandemic Estimated Fitness, Subgroup Analysis.

|  | Q1 | Q2 | Q3 | Q4 | Q5 |  |
| --- | --- | --- | --- | --- | --- | --- |
|  |  | IRR (95% CI) | IRR (95% CI) | IRR (95% CI) | IRR (95% CI) | P-value interaction |
| Sex |  |  |  |  |  |  |
| Men | Ref | 0.68 (0.43 – 1.07) | 0.49 (0.29 – 0.83) | 0.51 (0.30 – 0.87) | 0.52 (0.30 – 0.89) |  |
| Women | Ref | 0.84 (0.42 – 1.64) | 1.18 (0.64 – 2.18) | 1.04 (0.54 – 2.01) | 0.59 (0.26 – 1.37) | 0.17 |
| Smoking |  |  |  |  |  |  |
| Never | Ref | 0.57 (0.29 – 1.13) | 0.72 (0.39 – 1.34) | 0.52 (0.26 – 1.04) | 0.37 (0.18 – 0.77) |  |
| Current | Ref | 0.23 (0.05 – 1.08) | 0.63 (0.23 – 1.75) | 0.59 (0.20 – 1.71) | 0.35 (0.08 – 1.57) | 0.58 |
| Income |  |  |  |  |  |  |
| <1 million NOK | Ref | 0.72 (0.48 – 1.06) | 0.75 (0.51 – 1.12) | 0.69 (0.45 – 1.05) | 0.53 (0.33 – 0.86) |  |
| ≥1 million NOK | Ref | 0.68 (0.17 – 2.79) | - | 0.27 (0.05 – 1.58) | 0.41 (0.10 – 1.59) | 0.07 |
| Hypertension |  |  |  |  |  |  |
| No | Ref | 0.59 (0.33 – 1.07) | 0.69 (0.39 – 1.21) | 0.48 (0.26 – 0.89) | 0.37 (0.19 – 0.73) |  |
| yes | Ref | 0.84 (0.52 – 1.38) | 0.72 (0.42 – 1.24) | 0.88 (0.52 – 1.49) | 0.77 (0.43 – 1.40) | 0.28 |
| Diabetes |  |  |  |  |  |  |
| No | Ref | 0.62 (0.39 – 0.97) | 0.76 (0.49 – 1.16) | 0.64 (0.41 – 1.01) | 0.61 (0.38 – 0.99) |  |
| Yes | Ref | 1.16 (0.58 – 2.33) | 0.33 (0.10 – 1.14) | 0.86 (0.35 – 2.13) | - | 0.02 |
| Myocardial infarction |  |  |  |  |  |  |
| No | Ref | 0.65 (0.44 – 0.98) | 0.66 (0.43 – 0.99) | 0.66 (0.43 – 1.00) | 0.49 (0.30 – 0.79) |  |
| Yes | Ref | 1.89 (0.55 – 6.49) | 1.38 (0.35 – 5.48) | 0.91 (0.15 – 5.36) | 2.18 (0.49 – 9.72) | 0.33 |
| Cancer |  |  |  |  |  |  |
| No | Ref | 0.79 (0.52 – 1.20) | 0.73 (0.47 – 1.12) | 0.65 (0.41 – 1.03) | 0.49 (0.29 – 0.82) |  |
| Yes |  | 0.42 (0.16 – 1.09) | 0.52 (0.20 – 1.37) | 0.69 (0.27 – 1.73) | 0.79 (0.30 – 2.07) | 0.38 |

Abbreviations: IRR, incidence rate ratio; CI, confidence interval. IRR adjusted for age, sex, education, income, smoking, alcohol, diabetes, hypertension, myocardial infarction, angina, heart failure, stroke, and cancer.

**Table S5.** Adjusted Incidence Rate Ratio of Hospitalization Due to COVID-19 by Level of Pre-Pandemic Physical Activity Level, Subgroup Analysis.

|  | Inactive | Insufficiently active | Sufficiently active |  |
| --- | --- | --- | --- | --- |
|  |  | IRR (95% CI) | IRR (95% CI) | P-value interaction |
| Sex |  |  |  |  |
| Men | Ref | 0.79 (0.50 – 1.27) | 0.51 (0.34 – 0.76) |  |
| Women | Ref | 0.88 (0.46 – 1.70) | 0.83 (0.49 – 1.40) | 0.25 |
| Smoking |  |  |  |  |
| Never | Ref | 0.52 (0.25 – 1.09) | 0.52 (0.31 – 0.86) |  |
| Current | Ref | 1.69 (0.61 – 4.64) | 0.43 (0.12 – 1.50) | 0.35 |
| Body mass index |  |  |  |  |
| <25 kg/m^2^ | Ref | 0.66 (0.29 – 1.49) | 0.53 (0.27 – 1.03) |  |
| ≥25 kg/m^2^ | Ref | 0.87 (0.57 – 1.33) | 0.61 (0.43 – 0.87) | 0.35 |
| Income |  |  |  |  |
| <1 million NOK | Ref | 0.87 (0.59 – 1.28) | 0.62 (0.44 – 0.86) |  |
| ≥1 million NOK | Ref | 0.29 (0.03 – 2.81) | 0.36 (0.14 – 0.98) | 0.50 |
| Hypertension |  |  |  |  |
| No | Ref | 0.90 (0.49 – 1.68) | 0.78 (0.49 – 1.23) |  |
| yes | Ref | 0.79 (0.49 – 1.26) | 0.50 (0.32 – 0.76) | 0.76 |
| Diabetes |  |  |  |  |
| No | Ref | 0.81 (0.53 – 1.23) | 0.59 (0.42 – 0.84) |  |
| Yes | Ref | 0.88 (0.37 – 2.07) | 0.68 (0.33 – 1.41) | 0.90 |
| Myocardial infarction |  |  |  |  |
| No | Ref | 0.85 (0.57 – 1.25) | 0.56 (0.40 – 0.78) |  |
| Yes | Ref | 0.74 (0.17 – 3.16) | 1.52 (0.59 – 3.93) | 0.10 |
| Cancer |  |  |  |  |
| No | Ref | 0.89 (0.59 – 1.34) | 0.54 (0.38 – 0.77) |  |
| Yes |  | 0.57 (0.21 – 1.58) | 0.89 (0.43 – 1.86) | 0.11 |

Abbreviations: IRR, incidence rate ratio; CI, confidence interval. IRR adjusted for age, sex, body mass index, education, income, smoking, alcohol, diabetes, hypertension, myocardial infarction, angina, heart failure, stroke, and cancer.

**Table S6.** Adjusted Incidence Rate Ratio of Hospitalization due to COVID-19 by Pre-Pandemic estimated fitness levels, after excluding individuals with co-morbid conditions

| **eCRF** | **COVID-19 hospitalizations** | **IRR (95% CI)** |
| --- | --- | --- |
| Q1 | 41 | 1.00 (ref) |
| Q2 | 27 | 0.71 (0.44 – 1.16) |
| Q3 | 29 | 0.78 (0.49 – 1.26) |
| Q4 | 26 | 0.71 (0.43 – 1.17) |
| Q5 | 16 | 0.44 (0.24 – 0.80) |
|  |  |  |
| Least fit (≤20%) | 41 | 1.00 (ref) |
| Fit (>20%) | 98 | 0.67 (0.46 – 0.98) |
|  |  |  |
| Per MET | 139 | 0.84 (0.76 – 0.91) |
| Per mL/kg/min | 139 | 0.95 (0.92 – 0.97) |

Abbreviations: IRR, incidence rate ratio; CI, confidence interval. Q1: 28.2 for men and 23.7 for women Q2: 35.0 for men and 28.3 for women Q3: 38.7 for men and 30.9 for women Q4: 42.0 for men and 33.4 for women, Q5: 47.3 for men and 37.1 for women. Adjusted for age, sex, education, income, smoking, alcohol, diabetes, and hypertension. Individuals with baseline history of myocardial infraction, angina, stroke, heart failure, and cancer were excluded

**Table S7.** Adjusted Incidence Rate Ratio of Hospitalization due to COVID-19 by level of Pre-Pandemic Physical Activity, after excluding individuals with co-morbid conditions

| **Physical activity** | **COVID-19 hospitalizations** | **IRR (95% CI)** |
| --- | --- | --- |
| **3 groups^a^** |  |  |
| Inactive | 72 | 1.00 (ref) |
| Insufficiently active | 25 | 0.91 (0.57 – 1.45) |
| Sufficiently active | 42 | 0.55 (0.37 – 0.82) |
| **2 groups^b^** |  |  |
| Insufficiently active | 97 | 1.00 (ref) |
| Sufficiently active | 42 | 0.57 (0.39 – 0.82) |

Abbreviations: MET, Metabolic equivalent of task; IRR, incidence rate ratio; CI, confidence interval

^a^Inactive = 0 to 3.5 MET h/wk; insufficiently active = more than 3.5 to less than 7.5 MET hr/wk; and sufficiently active = 7.5 or more MET h/wk. ^b^Insufficiently active = 0 to less than 7.5 MET hr/wk; and sufficiently active 7.5 or more MET h/wk. Adjusted for age, sex, body mass index, education, income, smoking, alcohol, diabetes, and hypertension. Individuals with baseline history of myocardial infraction, angina, stroke, heart failure, and cancer were excluded.

**Table S8.** Adjusted Incidence Rate Ratio of Hospitalization due to COVID-19 by Pre-Pandemic estimated fitness levels, additionally adjusting for SARS CoV-2 vaccination status (modelled as time-dependent)

| **eCRF** | **COVID-19 hospitalizations** | **IRR (95% CI)** |
| --- | --- | --- |
| Q1 | 66 | 1.00 (ref) |
| Q2 | 45 | 0.73 (0.50 – 1.07) |
| Q3 | 40 | 0.67 (0.45 – 1.01) |
| Q4 | 38 | 0.66 (0.44 – 0.99) |
| Q5 | 29 | 0.52 (0.33 – 0.83) |
|  |  |  |
| Least fit (≤20%) | 66 | 1.00 (ref) |
| Fit (>20%) | 152 | 0.66 (0.48 – 0.89) |
|  |  |  |
| Per MET | 218 | 0.84 (0.78 – 0.91) |
| Per mL/kg/min | 218 | 0.95 (0.93 – 0.97) |

Abbreviations: IRR, incidence rate ratio; CI, confidence interval. Q1: 28.2 for men and 23.7 for women Q2: 35.0 for men and 28.3 for women Q3: 38.7 for men and 30.9 for women Q4: 42.0 for men and 33.4 for women, Q5: 47.3 for men and 37.1 for women. Adjusted for age, sex, education, income, smoking, alcohol, diabetes, hypertension, myocardial infarction, angina, heart failure, stroke, cancer, and vaccination status.

**Table S9.** Adjusted Incidence Rate Ratio of Hospitalization due to COVID-19 by level of Pre-Pandemic Physical Activity, additionally adjusting for SARS CoV-2 vaccination status (modelled as time-dependent)

| **Physical activity** | **COVID-19 hospitalizations** | **IRR (95% CI)** |
| --- | --- | --- |
| **3 groups^a^** |  |  |
| Inactive | 113 | 1.00 (ref) |
| Insufficiently active | 37 | 0.79 (0.54 – 1.15) |
| Sufficiently active | 68 | 0.56 (0.41 – 0.77) |
| **2 groups^b^** |  |  |
| Insufficiently active | 150 | 1.00 (ref) |
| Sufficiently active | 68 | 0.60 (0.45 – 0.81) |

Abbreviations: MET, Metabolic equivalent of task; IRR, incidence rate ratio; CI, confidence interval. ^a^Inactive = 0 to 3.5 MET h/wk; insufficiently active = more than 3.5 to less than 7.5 MET hr/wk; and sufficiently active = 7.5 or more MET h/wk. ^b^Insufficiently active = 0 to less than 7.5 MET hr/wk; and sufficiently active 7.5 or more MET h/wk. Adjusted for age, sex, body mass index, education, income, smoking, alcohol, diabetes, hypertension, myocardial infarction, angina, heart failure, stroke, cancer, and vaccination status.

**Table S10.** Hazard Ratios from Cox model for Hospitalization due to COVID-19 by Pre-Pandemic Estimated Cardiorespiratory Fitness and Physical Activity.

| **eCRF ^a^** | **COVID-19 hospitalizations** | **HR (95% CI)** |
| --- | --- | --- |
| Q1 | 66 | 1.00 (ref) |
| Q2 | 45 | 0.72 (0.49 – 1.06) |
| Q3 | 40 | 0.67 (0.45 – 1.00) |
| Q4 | 38 | 0.66 (0.44 – 0.99) |
| Q5 | 29 | 0.52 (0.32 – 0.82) |
| Per mL/kg/min | 218 | 0.95 (0.93 – 0.97) |
| Per MET | 218 | 0.84 (0.78 – 0.91) |
|  |  |  |
| **Least fit (≤20%)** | 66 | 1.00 (ref) |
| **Fit (>20%)** | 152 | 0.65 (0.48 – 0.88) |
|  |  |  |
| **Physical activity** |  |  |
| Inactive ^b^ | 113 | 1.00 (ref) |
| Insufficiently active ^c^ | 37 | 0.79 (0.54 – 1.15) |
| Sufficiently active ^d^ | 68 | 0.56 (0.41 – 0.77) |

Abbreviations: HR, hazard ratio; CI, confidence interval; MET, Metabolic equivalent of task. ^a^ Quintiles of VO_2peak_ in mL/kg/min for males and females, respectively: Q1: 28.2 for men and 23.7 for women, Q2: 35.0 for men and 28.3 for women, Q3: 38.7 for men and 30.9 for women, Q4: 42.0 for men and 33.4 for women, Q5: 47.3 for men and 37.1 for women. ^b^ = 0 to 3.5 MET h/wk; ^c^ = more than 3.5 to less than 7.5 MET h/wk; ^d^ = 7.5 or more MET h/wk.

HRs are adjusted for age, sex, education, income, smoking, alcohol, diabetes, hypertension, myocardial infarction, angina, heart failure, stroke, cancer, and vaccination status (time dependent).

**Table S11.** Hazard Ratios from Flexible Parametric Model (stpm2) for Hospitalization due to COVID-19 by Pre-Pandemic Estimated Cardiorespiratory Fitness and Physical Activity.

| **eCRF ^a^** | **COVID-19 hospitalizations** | **HR (95% CI)** |
| --- | --- | --- |
| Q1 | 66 | 1.00 (ref) |
| Q2 | 45 | 0.72 (0.49 – 1.06) |
| Q3 | 40 | 0.67 (0.45 – 1.00) |
| Q4 | 38 | 0.66 (0.44 – 0.99) |
| Q5 | 29 | 0.52 (0.33 – 0.82) |
| Per mL/kg/min | 218 | 0.95 (0.93 – 0.97) |
| Per MET | 218 | 0.84 (0.78 – 0.91) |
|  |  |  |
| **Least fit (≤20%)** | 66 | 1.00 (ref) |
| **Fit (>20%)** | 152 | 0.65 (0.48 – 0.88) |
|  |  |  |
| **Physical activity** |  |  |
| Inactive ^b^ | 113 | 1.00 (ref) |
| Insufficiently active ^c^ | 37 | 0.79 (0.54 – 1.15) |
| Sufficiently active ^d^ | 68 | 0.56 (0.41 – 0.77) |

Abbreviations: HR, hazard ratio; CI, confidence interval; MET, Metabolic equivalent of task. ^a^ Quintiles of VO_2peak_ in mL/kg/min for males and females, respectively: Q1: 28.2 for men and 23.7 for women, Q2: 35.0 for men and 28.3 for women, Q3: 38.7 for men and 30.9 for women, Q4: 42.0 for men and 33.4 for women, Q5: 47.3 for men and 37.1 for women. ^b^ = 0 to 3.5 MET h/wk; ^c^ = more than 3.5 to less than 7.5 MET h/wk; ^d^ = 7.5 or more MET h/wk.

HRs are adjusted for age, sex, education, income, smoking, alcohol, diabetes, hypertension, myocardial infarction, angina, heart failure, stroke, cancer, and vaccination status (time dependent).

**Table S12**. Competing Event (Death) Analyses for Hospitalization due to COVID-19 by Pre-Pandemic Estimated Cardiorespiratory Fitness and Physical Activity.

| **eCRF ^a^** | **COVID-19 hospitalizations** | **SHR (95% CI)** |
| --- | --- | --- |
| Q1 | 66 | 1.00 (ref) |
| Q2 | 45 | 0.72 (0.49 – 1.07) |
| Q3 | 40 | 0.67 (0.45 – 1.00) |
| Q4 | 38 | 0.66 (0.44 – 1.00) |
| Q5 | 29 | 0.53 (0.33 – 0.84) |
|  |  |  |
| **Physical activity** |  |  |
| Inactive ^b^ | 113 | 1.00 (ref) |
| Insufficiently active ^c^ | 37 | 0.79 (0.54 – 1.16) |
| Sufficiently active ^d^ | 68 | 0.57 (0.42 – 0.79) |

The competing event is death.

SHR, subdistribution hazard ratio are from Fine-Gray model and are adjusted for age, sex, education, income, smoking, alcohol, diabetes, hypertension, myocardial infarction, angina, heart failure, stroke, cancer, and vaccination status (time dependent).

**Table S13**. Competing Event (vaccination) Analyses for Hospitalization due to COVID-19 by Pre-Pandemic Estimated Cardiorespiratory Fitness and Physical Activity.

| **eCRF ^a^** | **COVID-19 hospitalizations** | **SHR (95% CI)** |
| --- | --- | --- |
| Q1 | 66 | 1.00 (ref) |
| Q2 | 45 | 0.72 (0.49 – 1.05) |
| Q3 | 40 | 0.69 (0.46 – 1.02) |
| Q4 | 38 | 0.66 (0.44 – 0.99) |
| Q5 | 29 | 0.55 (0.35 – 0.86) |
|  |  |  |
| **Physical activity** |  |  |
| Inactive ^b^ | 113 | 1.00 (ref) |
| Insufficiently active ^c^ | 37 | 0.81 (0.56 – 1.18) |
| Sufficiently active ^d^ | 68 | 0.58 (0.43 – 0.80) |

The competing event is vaccination.

SHR, subdistribution hazard ratio are from Fine-Gray model and are adjusted for age, sex, education, income, smoking, alcohol, diabetes, hypertension, myocardial infarction, angina, heart failure, stroke, cancer.

**Table S14**. Restricting to recent exposure keeping 01 Jan 2018 onward

| **eCRF ^a^** | **Participants** | **COVID-19 hospitalizations** | **IRR (95% CI)^1^** | **IRR (95% CI)^2^** | **HR (95% CI)^3^** |
| --- | --- | --- | --- | --- | --- |
| Q1 | 7076 | 45 | 1.00 (ref) | 1.00 (ref) | 1.00 (ref) |
| Q2 | 6895 | 23 | 0.53 (0.32 – 0.88) | 0.54 (0.32 – 0.89) | 0.54 (0.32 – 0.89) |
| Q3 | 6780 | 27 | 0.68 (0.43 – 1.09) | 0.69 (0.43 – 1.11) | 0.69 (0.43 – 1.11) |
| Q4 | 6760 | 22 | 0.57 (0.34 – 0.96) | 0.58 (0.34 – 0.97) | 0.58 (0.34 – 0.97) |
| Q5 | 6601 | 20 | 0.58 (0.33 – 1.00) | 0.55 (0.31 – 0.99) | 0.55 (0.31 – 0.97) |
|  |  |  |  |  |  |
| **Physical activity** |  |  |  |  |  |
| Inactive | 13,014 | 64 | 1.00 (ref) | 1.00 (ref) | 1.00 (ref) |
| Insufficiently active | 4820 | 29 | 1.21 (0.77 – 1.90) | 1.14 (0.72 – 1.81) | 1.16 (0.72 – 1.83) |
| Sufficiently active | 16,278 | 44 | 0.73 (0.49 – 1.08) | 0.71 (0.47 – 1.05) | 0.71 (0.47 – 1.06) |

IRR, incidence rate ratio from modified Poisson model

HR, hazard ratio from Cox model

^1^Adjusted for age, sex, education, income, smoking, alcohol, diabetes, hypertension, myocardial infarction, angina, heart failure, stroke, cancer, and vaccination status.

^2^Adjusted for age, sex, education, income, smoking, alcohol, diabetes, hypertension, myocardial infarction, angina, heart failure, stroke, cancer, and vaccination status (time dependent).

^3^Adjusted for age, sex, education, income, smoking, alcohol, diabetes, hypertension, myocardial infarction, angina, heart failure, stroke, cancer, and vaccination status (time dependent).

**Table S15**. Standardized Cumulative Incidence (CIF) of COVID-19 hospitalization by Pre-Pandemic Estimated Cardiorespiratory fitness, at end of follow-up.

| **eCRF ^a^** | **Standardized CIF (%)** | **Risk Ratios** | **Risk Difference**  **(percentage points)** |
| --- | --- | --- | --- |
| Q1 | 0.56 | Reference | Reference |
| Q2 | 0.41 | 0.73 | −0.15 |
| Q3 | 0.38 | 0.68 | −0.18 |
| Q4 | 0.37 | 0.66 | −0.19 |
| Q5 | 0.29 | 0.52 | −0.27 |
|  |  |  |  |
| **Physical activity** |  |  |  |
| Inactive ^b^ | 0.52 | Reference | Reference |
| Insufficiently active ^c^ | 0.41 | 0.79 | −0.11 |
| Sufficiently active ^d^ | 0.30 | 0.57 | −0.23 |

Cummulative Incidence Function (CIF) expressed as standardized absolute risk at the end of follow-up.

Risk ratio was calcaulted as CIF_k/CIF_reference.

Risk differene was calculated as CIF_k − CIF_reference, reported in percentage points. A negative risk difference indicated lower absolute risk than reference.

At the end of follow-up, the standardized cumulative incidence (standardized absolute risk) of hospitalization was 0.56% in quintile 1, decreasing across quintiles to 0.29% in quintile 5. Compared with quintile 1, quintile 5 had about a 48% lower absolute risk (Risk Ratio 0.52, Risk Difference –0.27%).

The model-based standardized cumulative incidence of COVID-19 hospitalization at study end was 0.52% in inactive participants, 0.41% in insufficiently active, and 0.30% in sufficiently active participants. Compared with inactive participants, sufficiently active participants had a lower absolute risk (risk difference −0.23 percentage points) and approximately 43% lower risk (Risk Ratio 0.57).

**Table S16.** Association between accidental death and pre-pandemic estimated cardiorespiratory fitness and leisure-time physical activity.

|  | **Accidental death** | **IRR (95% CI)** | |
| --- | --- | --- | --- |
| **eCRF ^a^** | **Number** | **Model 1** | **Model 2** |
| Q1 | 10 | 1.00 (ref) | 1.00 (ref) |
| Q2 | 6 | 0.65 (0.24 – 1.78) | 0.65 (0.24 – 1.77) |
| Q3 | 11 | 1.32 (0.56 – 3.12) | 1.38 (0.59 – 3.23) |
| Q4 | 7 | 0.87 (0.33 – 2.28) | 0.95 (0.36 – 2.47) |
| Q5 | 5 | 0.71 (0.26 – 1.96) | 0.81 (0.30 – 2.21) |
| Per mL/kg/min | 39 | 1.01 (0.95 – 1.06) | 1.01 (0.97 – 1.07) |
|  |  |  |  |
| **Least fit (≤20%)** | 10 | 1.00 (ref) | 1.00 (ref) |
| **Fit (>20%)** | 29 | 0.90 (0.44 – 1.81) | 0.94 (0.47 – 1.88) |
|  |  |  |  |
| **Leisure-time physical activity** |  |  |  |
| Inactive ^b^ | 18 | 1.00 (ref) | 1.00 (ref) |
| Insufficiently active ^c^ | 9 | 1.19 (0.53 – 2.70) | 1.22 (0.54 – 2.77) |
| Sufficiently active ^d^ | 12 | 0.70 (0.33 – 1.47) | 0.76 (0.36 – 1.60) |
| Per MET | 39 | 0.98 (0.93 – 1.03) | 0.98 (0.93 – 1.03) |

Abbreviations: IRR, incidence rate ratio; CI, confidence interval; MET, Metabolic equivalent of task. ^a^ Quintiles of VO_2peak_ in mL/kg/min for males and females, respectively: Q1: 28.2 for men and 23.7 for women, Q2: 35.0 for men and 28.3 for women, Q3: 38.7 for men and 30.9 for women, Q4: 42.0 for men and 33.4 for women, Q5: 47.3 for men and 37.1 for women. ^b^ = 0 to 3.5 MET h/wk; ^c^ = more than 3.5 to less than 7.5 MET h/wk; ^d^ = 7.5 or more MET h/wk. Model 1: Adjusted for age, sex, education, income, smoking, alcohol. Model 2: Adjusted for age, sex, education, income, smoking, alcohol, diabetes, hypertension, myocardial infarction, angina, heart failure, stroke, and cancer.

**Table S17.** Hazard Ratios from Cox Model for Accidental Deaths by Pre-Pandemic Estimated Cardiorespiratory Fitness and Physical Activity.

| **eCRF ^a^** | **Accidental deaths** | **HR (95% CI)** |
| --- | --- | --- |
| Q1 | 10 | 1.00 (ref) |
| Q2 | 6 | 0.65 (0.24 – 1.77) |
| Q3 | 11 | 1.38 (0.59 – 3.23) |
| Q4 | 7 | 0.95 (0.36 – 2.47) |
| Q5 | 5 | 0.81 (0.30 – 2.21) |
|  |  |  |
| **Least fit (≤20%)** | 10 | 1.00 (ref) |
| **Fit (>20%)** | 29 | 0.94 (0.47 – 1.88) |
|  |  |  |
| **Physical activity** |  |  |
| Inactive ^b^ | 18 | 1.00 (ref) |
| Insufficiently active ^c^ | 9 | 1.34 (0.60 – 3.01) |
| Sufficiently active ^d^ | 12 | 0.85 (0.41 – 1.76) |

Abbreviations: HR, hazard ratio; CI, confidence interval; ^a^ Quintiles of VO_2peak_ in mL/kg/min for males and females, respectively: Q1: 28.2 for men and 23.7 for women, Q2: 35.0 for men and 28.3 for women, Q3: 38.7 for men and 30.9 for women, Q4: 42.0 for men and 33.4 for women, Q5: 47.3 for men and 37.1 for women. ^b^ = 0 to 3.5 MET h/wk; ^c^ = more than 3.5 to less than 7.5 MET h/wk; ^d^ = 7.5 or more MET h/wk.

HRs are adjusted for age, sex, education, income, smoking, alcohol, diabetes, hypertension, myocardial infarction, angina, heart failure, stroke, and cancer.
